# Synthesis, DNA Binding, and Anticancer Properties of Bis-Naphthalimide Derivatives with Lysine-Modified Polyamine Linkers

**DOI:** 10.3390/molecules23020266

**Published:** 2018-01-29

**Authors:** Yu Huang, Chun-Xia Wu, Yu Song, Min Huang, Da-Nian Tian, Xin-Bin Yang, Yan-Ru Fan

**Affiliations:** 1 Key Laboratory of Hui Ethnic Medicine Modernization, Ministry of Education, Ningxia Engineering and Technology Research Centre of Hui Medicine Modernization, College of Pharmacy, Ningxia Medical University, Yinchuan 750004, China; wuchunxiapharmacy@163.com (C.-X.W.); songyuouc@163.com (Y.S.); 2 College of Public Health, Ningxia Medical University, Yinchuan 750004, China; huangmin@163.com (M.H.); tiandanian@163.com (D.-N.T.); 3 Rongchang Campus, Southwest University, Chongqing 402460, China; yangxbqq@126.com

**Keywords:** bis-naphthalimide derivatives, DNA binding, cytotoxicity

## Abstract

A series of bis-naphthalimide derivatives with different diamine linkers were designed and synthesized. All of the synthesized bis-naphthalimide derivatives were characterized by NMR and HRMS spectra. The binding ability between the compounds and CT DNA was evaluated by using UV–Vis titration experiments. The bis-naphthalimide compound with an ethylenediamine linker showed the largest binding constant with CT DNA. Hence, it was used as the model compound to study the DNA binding selectivity by UV–Vis titration aiming at different DNA duplexes. As a result, this compound showed binding preference to AT-rich duplexes. The DNA binding modes of the compounds were also measured by viscosity titration. The cytotoxicity of the compounds was evaluated by MTT assay. Compounds with 1,6-diaminohexane or 1,4-phenylenedimethanamine linkers showed higher cytotoxicity compared with other bis-naphthalimide derivatives.

## 1. Introduction

Naphthalimide (1*H*-benzo[de]isoquinoline-1,3-(2*H*)-diones), a kind of flat heteroaromatic polycyclic amide, has been studied for decades due to its potential for the development of antitumor drugs [1]. Though some of its derivatives were approved for clinical trials, all of the trials were terminated due to the toxic side effects [2,3,4,5,6,7]. Therefore, to improve the antitumor activity and reduce the side effects, modifications on the naphthalimide structure have been carried out in recent decades; some naphthalimide derivatives with different side chains, aromatic ring systems, and substituents on the ring have been designed and synthesized [8,9,10,11].

Dimerization of naphthalimide is one of the generally used methods to improve its antitumor efficiency. The dimeric naphthalimide derivatives usually exhibit higher activity than the monomeric ones. The bis-naphthalimide Elinafide, which exhibits high activity against a series of human xenograft models, is a well-known bis-intercalator in all the naphthalimide derivatives [12]. Dimerization has also been employed to link the two pharmacophores [13]. Among all types of spacers, besides Tröger’s base moieties [14,15], polyamine spacers have been widely developed in the construction of bis-intercalators which exhibited high DNA binding ability and excellent anticancer activity [16,17,18,19,20]. Some other studies in the literature also reported the influence of bis-naphthalimide derivatives with polyamine spacers on DNA [21,22,23]. Lin et al. reported a bis-naphthalimide derivative linked by spermidine, which showed an IC_50_ value of 0.15 and 1.64 μM towards Caco-2 and HT-29 colon adenocarcinoma cells and induced significant DNA damage [24]. Other results from Li and co-workers showed that an *N*-(2-hydroxyethyl)piperazine-modified bis-naphthalimide derivative showed better cytotoxic activity than the control drug, amonafide [25]. This compound also exhibited fluorescence enhancement upon binding with DNA.

To improve the anticancer ability, amino acids with chiral side chains were combined into the structure of naphthalimide derivatives. Qian et al. reported some naphthalimide derivatives with flexible leucine side chains which exhibited the IC_50_ values of 10^−6^–10^−5^ M against HeLa, A549, P388, HL-60, MCF-7, HCT-8, and A375 cancer cell lines in vitro [26]. In addition, the chirality of the amino side chains is important for the naphthalimide derivatives. Yang et al. reported that the S-enantiomers showed better DNA binding activity and DNA photocleavage ability than did the R-enantiomers [27].

Herein, we report the synthesis of novel bis-naphthalimide derivatives modified by lysine and different diamine linkers. The DNA binding ability and cytotoxicity of these compounds were systematically studied. All of the compounds exhibited high DNA binding ability and showed binding preference to AT-rich (adenine and thymine) duplexes. However, the cytotoxicity of these compounds was not so good.

## 2. Results and Discussion

### 2.1. Chemistry

**Scheme 1:**
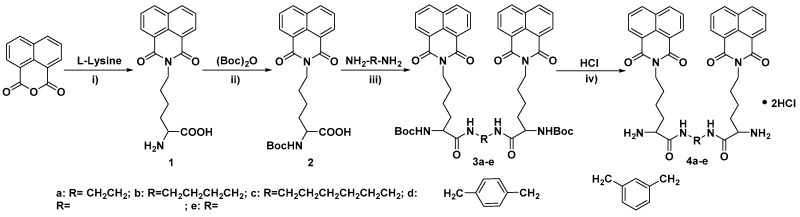
The synthesis route of bis-naphthalimide derivatives. Reagents and conditions: (i) ethanol, reflux 30 h; (ii) Na_2_CO_3_, THF/Water (2:1), room temperature; (iii) triethylamine (Et_3_N), 4-dimethylaminopyridine (DMAP), 1-hydroxybenzotriazole (HOBt), dicyclohexylcarbodiimide (DCC), dichloromethane; (iv) ethanol.

All of the bis-naphthalimide derivatives were synthesized from 1,8-naphthalic anhydride as shown in Scheme 1. Firstly, 1,8-naphthalic anhydride was refluxed with l-lysine in ethanol to obtain compound **1**. Then, compound **1** was reacted with di-*tert*-butyl dicarbonate to protect the amino group. After that, the carboxyl group was condensed with different diamines by a DCC (Dicyclohexylcarbodiimide) coupling reaction. Finally, the bis-naphthalimide derivatives were obtained by deprotection of the Boc group.

### 2.2. UV–Vis Titration

**Figure 1:**
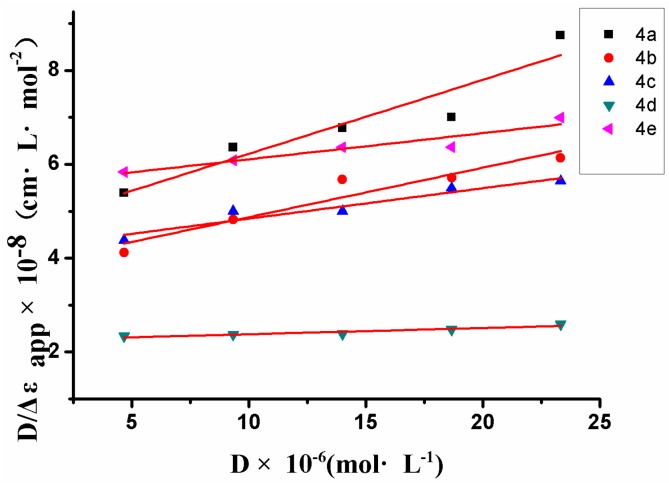
*D*/*Δε_app_* vs. *D* plot of bis-naphthalimide derivative **4** (50 μM) with CT DNA in PBS buffer (50.0 mM, pH = 7.4).

According to our laser scanning confocal microscopy experiments of other bis-naphthalimide derivatives, some of these active compounds reached the nucleus of the cell (unpublished results). Hence, it was necessary to evaluate the DNA binding ability of the synthesized bis-naphthalimide derivatives **4a**–**e**. The DNA binding abilities of the bis-naphthalimide derivatives and mononaphthalimide derivative **10** were evaluated by UV–Vis titration experiments, which was a useful method to calculate the DNA binding constants in vitro. After Calf Thymus DNA (CT DNA) was added to the phosphate buffer solution containing different compounds, all of the solutions exhibited a slight hypochromism without any significant spectral shift. The binding constants of the compounds were calculated by using a plot of *D*/*Δε_app_* versus *D*, as shown in Figure 1 and Table 1. Bis-naphthalimide derivative **4a** with the shortest linker showed the largest binding constant (3.40 × 10^4^ L/mol). The binding constants decreased with the linker length from ethylenediamine, to 1,4-butanediamine, to 1,6-hexanediamine. Compounds **4d** and **4e** with rigid bis(aminomethyl)benzene linkers were the weakest DNA binding reagents. The rigidity and length of the linkers influenced the binding ability of the bis-naphthalimide derivatives.

### 2.3. DNA Binding Selectivity

**Figure 2:**
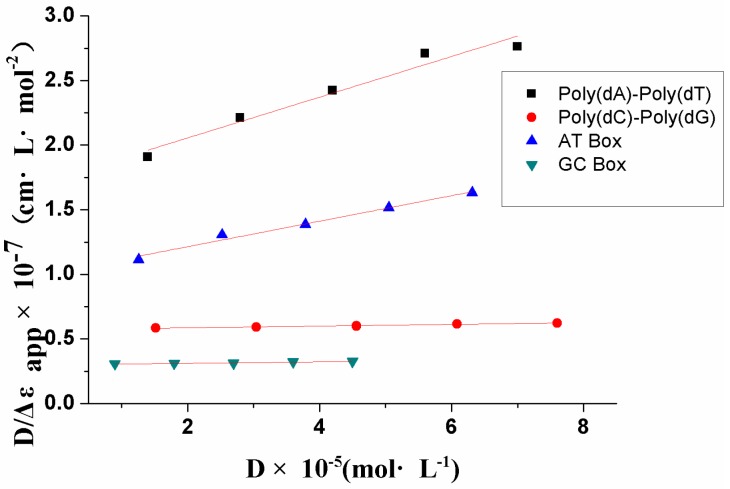
*D*/*Δε_app_* vs. *D* plot of bis-naphthalimide derivative **4a** (50 μM) with different DNA duplexes in PBS buffer (50.0 mM, pH = 7.4).

As compound **4a** exhibited the largest DNA binding ability among all of the bis-naphthalimide derivatives, it was used as the model compound to study the DNA binding selectivity. The phosphate buffer solution of compound **4a** was titrated using four different DNA duplexes: [Poly(dA)-Poly(dT)], [Poly(dG)-Poly(dC)], AT Box [Poly(dTATAAT)-Poly(dATATTA)], and GC Box [Poly(dGGGCGG)-Poly(dCCCGCC)], respectively. The *D*/*Δε_app_* vs. *D* plot of the model compound was recorded, as shown in Figure 2. The binding constant of compound **4a** with different DNA duplexes was calculated. As shown in Table 2, the bis-naphthalimide derivative **4a** showed a tendency to bind with AT-rich duplexes. The binding constants of **4a** with AT-rich duplexes [Poly(dA)-Poly(dT)] and AT Box were higher than those with the reference duplexes [Poly(dG)-Poly(dC)] and GC Box. The binding constant of the hybrid AT box was similar to that of [Poly(dA)-Poly(dT)], which meant that the compound **4a** preferred to bind with AT-rich duplexes, no matter how hybridized the AT-rich duplexes were. The binding selectivity of the bis-naphthalimide derivatives was in accordance with similar compounds [28].

### 2.4. Viscosity Studies

**Figure 3:**
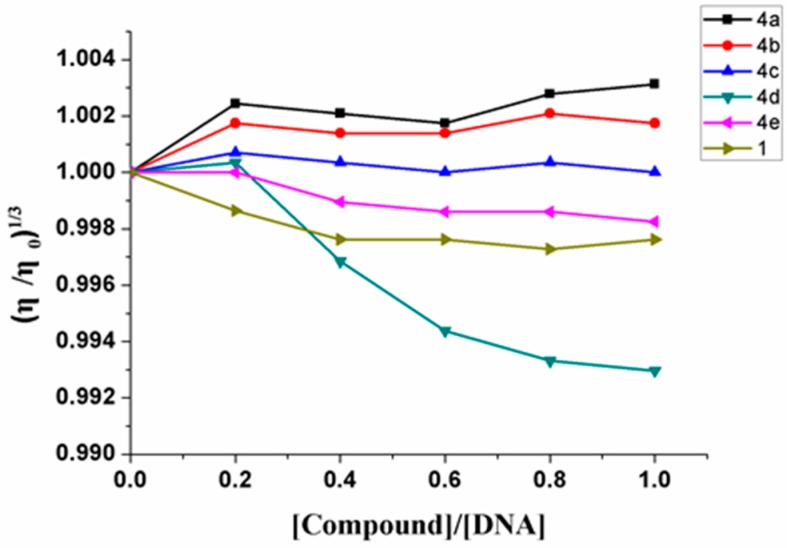
DNA viscosity titration of different bis-naphthalimide compounds **4a**–**e** at 35 °C in 25 mL phosphate buffer (50.0 mM, pH = 7.4). The concentration of DNA was 0.56 mM and the ratios between bis-naphthalimide compounds and DNA were 0.2, 0.4, 0.6, 0.8, and 1.0, respectively.

Although the UV–Vis titration data provided necessary information on the binding ability of bis-naphthalimide derivative, the detailed binding mode cannot be given by generally used optical photophysical methods. A viscosity experiment is a useful method and can provide critical information on different binding modes. Generally speaking, in classical intercalation binding mode, the intercalators insert into the base pairs of the DNA duplexes and extend the length of DNA, which significantly increases the viscosity of the DNA solution [29]. In partial and/or nonclassical intercalation binding mode, the length of DNA duplexes is reduced, which induces a decrease in the viscosity of the DNA solution [30]. As shown in Figure 3, the viscosity of the DNA solution increased after the addition of bis-naphthalimide derivatives **4a** and **4b** but decreased after the addition of compounds **4c**–**e** and **1**. These results showed that compounds **4a** and **4b** were classical DNA intercalators. Compounds **4c**–**e** and **1** bind with DNA through the partial intercalation binding mode. The rigidity of the linker significantly influenced the binding modes of the different bis-naphthalimide derivatives. To our surprise, the mononaphthalimide compound **1** exhibited a partial intercalation binding mode, which might be due to the electrostatic binding of the amino group interrupting the intercalation of the naphthalimide group. The weak binding of compound **4c** might have the same cause.

### 2.5. Cytotoxicity Assay

The cytotoxicity of bis-naphthalimide derivatives against human cancer cell lines EC109 and BGC823 were evaluated by MTT assay (Table 3). 5-Fluorouracil was tested as the reference compound. After the EC109 cell line and bis-naphthalimide derivatives were incubated for 24 h, compounds **4c** and **4d** (with longer linkers) showed higher cytotoxicity compared with other bis-naphthalimide derivatives. Hence, compounds **4c** and **4e** were used to test the cytotoxicity against the BGC823 cell line. According to the results, the bis-naphthalimide derivative **4e** showed moderate antitumor ability with an IC_50_ value of 77.99 μmol/L toward the BGC823 cell line, which was lower than that of 5-Fluorouracil.

### 2.6. Morphology Observation

**Figure 4:**
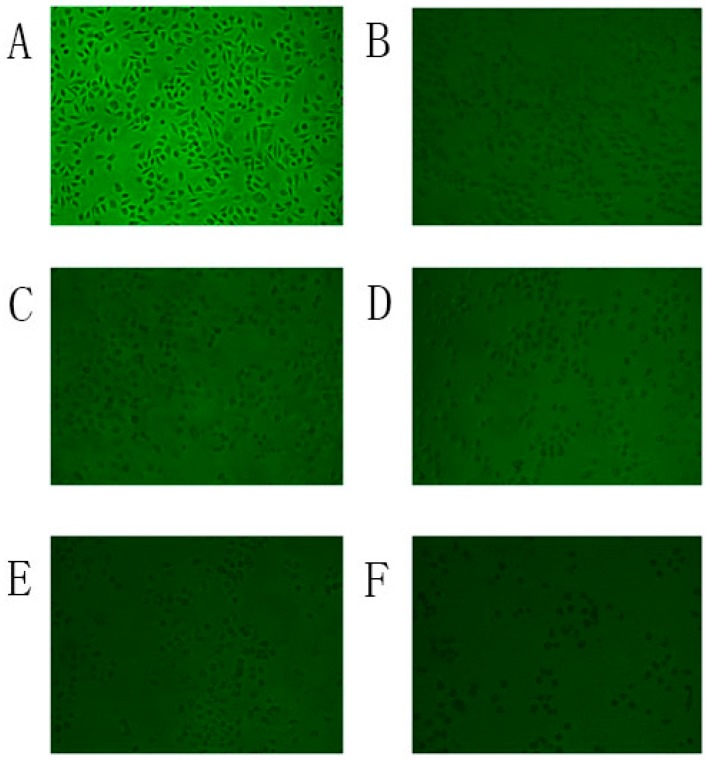
Morphological changes in BGC823 cells. The cells were treated with **4d** (**A**) Control; (**B**) 12.5 μM; (**C**) 25 μM; (**D**) 50 μM; (**E**) 100 μM; (**F**) 200 μM for 48 h and observed under a phase contrast microscope.

According to the results of the MTT assay, compound **13d** was used to study the morphological alterations of the BGC823 cell line in the absence and presence of bis-naphthalimide derivatives. In the absence of compound **4d**, the cancer cell line showed adherent growth with normal size and shape (Figure 4A). When cancer cells were incubated with compound **4d**, the cell counts dropped with the increasing concentration of compound **4d** (Figure 4B–F). The cancer cells became small and round at higher drug concentrations.

## 3. Materials and Methods

### 3.1. Chemistry

All of the chemical reagents and solvents were of analytic grade and obtained from commercial sources. CT DNA were from Sigma-Aldrich (St. Louis, MO, USA) and other DNA duplexes were from Sangon Biotech (Shanghai, China). UV–Vis spectra were measured on a Shimadzu UV-2501 spectrophotometer (Kyoto, Japan) at room temperature. ^1^H-NMR and ^13^C-NMR spectra were recorded on a Bruker AVIII 400 spectrometer (Fällanden, Switzerland), with tetramethylsilane (TMS) as an internal standard. Mass spectra were recorded on a Shimadzu LCMS-IT-TOF (Kyoto, Japan).

#### 3.1.1. Synthesis of *N*-Epsilon-1,8-naphthalimido-lysine (**1**)

1,8-Naphthalic anhydride (5.00 g, 25.23 mmol) was dissolved in 500 mL of ethanol. After the addition of l-Lysine (6.92 g, 37.88 mmol), the mixture was refluxed for 30 h. Then, the insoluble solid was removed by filtration while the solvent was hot. Another 500 mL of ethanol was added into the filtrate and the mixture was stored overnight. After filtering the insoluble solid, the ethanol was removed under reduced pressure to obtain the crude product. Hot water was added to the crude product, and the mixture solution was then stirred for 5 min. After the insoluble solid was filtered, the filtrate was cooled to room temperature. A yellow crystal crystallized from the water solution. The yield of *N*-epsilon-1,8-naphthalimido-lysine (**1**) was 60.0%. ^1^H-NMR (400 MHz, D_2_O) δ: 8.14 (d, *J* = 7.2 Hz, 2H, naphthalene-H), 7.88 (d, *J* = 8.0 Hz, 2H, naphthalene-H), 7.45 (t, *J* = 7.8 Hz, 2H, naphthalene-H), 5.40 (q, *J* = 5.32 Hz, 1H, NH_2_CH), 3.06 (t, *J* = 7.6 Hz, 2H, NCH_2_), 1.91–2.31 (m, 2H, CHCH_2_), 1.68–1.77 (m, 2H, NCH_2_CH_2_), 1.31–1.45 (m, 2H, CHCH_2_CH_2_). ^13^C-NMR (100 MHz, D_2_O) δ: 175.66, 165.25, 135.05, 131.48, 130.60, 126.81, 120.57, 62, 46, 54.70, 39.21, 28.63, 26.50, 23.19.

#### 3.1.2. Synthesis of *N*-Alpha-(*tert*-Butoxycarbonyl)-*N*-epsilon-1,8-naphthalimido-lysine (**2**)

To 300 mL of a THF/water (2:1) mixture solution of *N*-epsilon-1,8-naphthalimido-lysine (**1**) (6.60 g, 20.26 mmol), sodium carbonate (38.50 g) was added. The mixture solution was stirred in an ice-water bath until the temperature was cooled to 0 °C. Then, 30 mL THF solution of di-*tert*-butyl dicarbonate (4.85 g, 22.27 mmol) was added dropwise into the flask. After the mixture was stirred overnight at room temperature, the mixture was filtered and the filtrate was condensed under reduced pressure to obtain a water solution. The water solution was extracted by ethyl acetate (60 mL) three times. Then, the water solution was adjusted to pH 3 and extracted by methylene chloride (90 mL) three times. The organic layer was combined and washed with brine (30 mL), then dried with sodium sulfate. After filtration, the solvent was evaporated in vacuo to obtain the oil crude product. The pure oil product was obtained by silica gel column chromatography using petrol ether/ethyl acetate (1:2, *v*/*v*). The yield of *N*-alpha-(*tert*-Butoxycarbonyl)*-N*-epsilon-1,8-naphthalimido-lysine (**2**) was 83.5%. ^1^H-NMR (400 MHz, CDCl_3_) δ: 8.61 (d, *J* = 7.2 Hz, 2H, naphthalene-H), 8.24 (d, *J* = 8.0 Hz, 2H, naphthalene-H), 7.77 (t, *J* = 7.8 Hz, 2H, naphthalene-H), 5.76 (q, *J* = 5.12 Hz, 1H, BocNHCH), 4.51 (s, 1H, BocNH), 3.06 (d, *J* = 7.6 Hz, 2H, NCH_2_), 2.17–2.37 (m, 2H, CHCH_2_), 1.43–1.52 (m, 2H, NCH_2_CH_2_), 1.35 (s, 9H, Boc), 1.30 (t, *J* = 5.2 Hz, 2H, CHCH_2_CH_2_). ^13^C-NMR (100 MHz, CDCl_3_) δ: 164.00, 134.51, 131.99, 131.77, 128.52, 127.17, 122.34, 53.11, 40.42, 29.77, 28.61, 28.48, 23.74.

#### 3.1.3. Synthesis of **3**

In a 100 mL flask, *N*-alpha-(*tert*-Butoxycarbonyl)*-N*-epsilon-1,8-naphthalimido-lysine (**2**) (1.50 g, 3.50 mmol), trimethylamine (0.95 g, 9.60 mmol), 4-dimethylaminopyridine (DMAP, 0.39 g, 3.20 mmol), and different diamines (1.60 mmol) were dissolved in 50 mL methylene chloride. After stirring for 5 min, 1-hydroxybenzotriazole (HOBt, 0.52 g, 3.85 mmol) was added into the mixture. The mixture was stirred in an ice-water bath for 0.5 h. Then dicyclohexylcarbodiimide (DCC, 0.67 g, 3.50 mmol) was added into the mixture in batches. The mixture was warmed to room temperature and stirred overnight. Then, the mixture was washed with 5% citric acid solution (50 mL) and saturated sodium bicarbonate solution (50 mL). After drying with sodium sulfate, the solvent was evaporated in vacuo to obtain the crude product. After purification by silica gel column chromatography using petrol ether/ethyl acetate (1:3, *v*/*v*), the pure oil product was obtained.

*Bis-(N-alpha-(tert-Butoxycarbonyl)-N-epsilon-1,8-naphthalimido-lysyl) ethylenediamine* (**3a**). Yield 70.2%. ^1^H-NMR (400 MHz, CDCl_3_) δ: 8.41 (d, *J* = 7.2 Hz, 4H, naphthalene-H), 8.18 (d, *J* = 8.0 Hz, 4H, naphthalene-H), 7.68 (t, *J* = 7.8 Hz, 4H, naphthalene-H), 7.02 (s, 2H, CONH), 5.66 (t, *J* = 7.4 Hz, 2H, BocNHCH), 4.59 (s, 2H, BocNH), 3.32–3.49 (m, 4H, CONHCH_2_), 3.01–3.06 (m, 4H, NCH_2_), 2.27–2.28 (m, 4H, CHCH_2_), 1.46–1.57 (m, 4H, NCH_2_CH_2_), 1.36 (s, 18H, Boc), 1.25 (s, 4H, CHCH_2_CH_2_). ^13^C-NMR (100 MHz, CDCl_3_) δ: 170.70, 164.38, 156.09, 134.30, 131.74, 131.68, 128.50, 127.10, 122.56, 77.48, 77.16, 76.85, 54.86, 40.26, 39.22, 29.91, 28.48, 28.16, 23.87. HR-MS *m*/*z* (ESI), Calcd for C_48_H_56_N_6_O_10_ [M + H]^+^ 877.4141, found 877.4095.

*Bis-(N-alpha-(tert-Butoxycarbonyl)-N-epsilon-1,8-naphthalimido-lysyl)-1,4-butanediamin* (**3b**). Yield 73.5%. ^1^H-NMR (400 MHz, CDCl_3_) δ: 8.29 (d, *J* = 7.2 Hz, 4H, naphthalene-H), 8.19 (d, *J* = 8.0 Hz, 4H, naphthalene-H), 7.71 (t, *J* = 7.8 Hz, 4H, naphthalene-H), 6.32 (s, 2H, CONH), 5.53 (s, 2H, BocNHCH), 4.55 (s, 2H, BocNH), 3.26–3.30 (m, 4H, CONHCH_2_), 2.99–3.03 (m, 4H, NCH_2_), 2.15 (t, *J* = 7.6 Hz, 4H, CHCH_2_), 1.42–1.52 (m, 8H, CONHCH_2_CH_2_, NCH_2_CH_2_), 1.36 (s, 18H, Boc), 1.25 (t, *J* = 7.2 Hz, 4H, CHCH_2_CH_2_). ^13^C-NMR (100 MHz, CDCl_3_) δ: 169.76, 164.34, 156.11, 134.37, 134.30, 131.87, 131.77, 131.69, 128.48, 127.17, 127.12, 122.55, 77.48, 77.16, 76.84, 54.81, 40.27, 39.58, 29.79, 28.49, 28.13, 26.65, 23.85. HR-MS *m*/*z* (ESI), Calcd for C_50_H_60_N_6_O_10_ [M + H]^+^ 905.444, found 905.4350.

*Bis-(N-alpha-(tert-Butoxycarbonyl)-N-epsilon-1,8-naphthalimido-lysyl)-1,6-hexylenediamine* (**3c**). Yield 71%. ^1^H-NMR (400 MHz, CDCl_3_) δ: 8.55 (d, *J* = 7.2 Hz, 4H, naphthalene-H), 8.22 (d, *J* = 8.0 Hz, 4H, naphthalene-H), 7.75 (t, *J* = 7.8 Hz, 4H, naphthalene-H), 6.14 (s, 2H, CONH), 5.25 (t, *J* = 6.88 Hz, 2H, BocNHCH), 4.53 (s, 2H, BocNH), 2.59–3.02 (m, 8H, NCH_2_, CONHCH_2_), 2.11–2.15 (m, 4H, CHCH_2_), 1.39–1.45 (m, 8H, CONHCH_2_CH_2_, NCH_2_CH_2_), 1.36 (s, 18H, Boc), 1.20–1.33 (m, 4H, CHCH_2_CH_2_), 1.16–1.18 (m, 4H, CONHCH_2_CH_2_CH_2_). ^13^C-NMR (100 MHz, CDCl_3_) δ: 169.41, 164.33, 156.11, 134.38, 134.17, 131.81, 131.64, 131.60, 128.51, 127.15, 127.08, 122.73, 79.07, 54.42, 40.15, 37.89, 29.62, 28.50, 28.18, 24.45, 23.75. HR-MS *m*/*z* (ESI), Calcd for C_52_H_64_N_6_O_10_ [M + H]^+^ 933.4757, found 933.4642.

*Bis-(N-alpha-(tert-Butoxycarbonyl)-N-epsilon-1,8-naphthalimido-lysyl)-1,4-phenylenedimethan-amine* (**3d**). Yield 50.8%. ^1^H-NMR (400 MHz, CDCl_3_) δ: 8.57 (d, *J* = 7.2 Hz, 4H, naphthalene-H), 8.21 (d, *J* = 8.0 Hz, 4H, naphthalene-H), 7.74 (t, *J* = 7.8 Hz, 4H, naphthalene-H), 7.26 (s, 2H, benzene-H), 7.19 (s, 2H, benzene-H), 6.24 (s, 2H, CONH), 5.65 (q, *J* = 5.52 Hz, 2H, BocNHCH), 4.51 (s, 2H, BocNH), 4.37–4.48 (m, 4H, xylyl-H), 2.97–2.31 (m, 4H, NCH_2_), 2.23–2.31 (m, 4H, CHCH_2_), 1.48 (t, *J* = 5.2 Hz, 4H, NCH_2_CH_2_), 1.35 (s, 18H, Boc), 1.25 (s, 4H, CHCH_2_CH_2_). ^13^C-NMR (100 MHz, CDCl_3_) δ: 169.52, 164.38, 156.07, 137.52, 134.49, 131.97, 131.71, 128.49, 128.17, 127.19, 122.41, 54.82, 43.57, 40.22, 29.82, 28.56, 28.47, 28.20, 23.80. HR-MS *m*/*z* (ESI), Calcd for C_54_H_60_N_6_O_10_ [M + Na]^+^ 975.4263, found 975.4187.

*Bis-(N-alpha-(tert-Butoxycarbonyl)-N-epsilon-1,8-naphthalimido-lysyl)-1,3-phenylenedimethan-amine* (**3e**). Yield 74.1%. ^1^H-NMR (400 MHz, CDCl_3_) δ: 8.46 (d, *J* = 7.2 Hz, 4H, naphthalene-H), 8.08 (d, *J* = 8.0 Hz, 4H, naphthalene-H), 7.61 (t, *J* = 7.8 Hz, 4H, naphthalene-H), 7.29 (s, 1H, benzene-H), 7.26 (s, 1H, benzene-H), 7.13 (d, *J* = 7.88 Hz, 2H, benzene-H), 6.54 (s, 2H, CONH), 5.66 (t, *J* = 7.4, 2H, BocNHCH), 4.62 (s, 2H, BocNH), 4.37–4.55 (m, 4H, xylyl-H), 3.00–3.06 (m, 4H, NCH_2_), 2.25–2.31 (m, 4H, CHCH_2_), 1.51 (t, *J* = 5.8 Hz, 4H, NCH_2_CH_2_), 1.48 (s, 4H, CHCH_2_CH_2_), 1.37 (s, 18H, Boc). ^13^C-NMR (100 MHz, CDCl_3_) δ: 169.64, 164.30, 156.12, 138.93, 134.22, 131.70, 131.52, 128.79, 128.29, 127.03, 126.96, 126.65, 122.33, 79.04, 77.48, 77.16, 76.84, 54.88, 43.57, 40.27, 29.82, 28.47, 28.30, 23.92. HR-MS *m*/*z* (ESI), Calcd for C_54_H_60_N_6_O_10_ [M + Na]^+^ 975.4263, found 975.4223.

#### 3.1.4. Synthesis of **4**

In a 100 mL flask, compound **3** (0.25 mmol) was dissolved in 15 mL ethanol. A quantity of 30 mL HCl ethanol solution was added under stirring. The mixture was stirred at room temperature overnight. The solvent was evaporated in vacuo to obtain the crude product. After washing with methyl *tert*-butyl ether, a white powder was obtained.

*Bis-(N-epsilon-1,8-naphthalimido-lysyl) ethylenediamine* (**4a**). Yield 85.5%. ^1^H-NMR (400 MHz, D_2_O) δ: 8.21 (d, *J* = 7.2 Hz, 4H, naphthalene-H), 8.07 (d, *J* = 8.0 Hz, 4H, naphthalene-H), 7.58 (t, *J* = 7.8 Hz, 4H, naphthalene-H), 5.53 (q, *J* = 5.04 Hz, 2H, BocNHCH), 3.23–3.36 (m, 4H, NCH_2_), 2.93 (t, *J* = 7.8 Hz, 4H, CHCH_2_), 1.97–2.23 (m, 4H, NCH_2_CH_2_), 1.49–1.71 (m, 4H, CHCH_2_CH_2_), 1.32–1.34 (m, 4H, CONHCH_2_CH_2_). ^13^C-NMR (100 MHz, D_2_O) δ: 175.20, 165.27, 135.51, 131.67, 131.08, 127.41, 126.97, 120.76, 54.02, 39.37, 39.16, 29.58, 27.21, 26.43, 22.66, 16.77. HR-MS *m*/*z* (ESI), Calcd for C_38_H_40_N_6_O_6_ [M + H]^+^ 677.3082, found 677.3036.

*Bis-(N-epsilon-1,8-naphthalimido-lysyl)-1,4-butanediamin* (**4b**). Yield 79.2%. ^1^H-NMR (400 MHz, D_2_O) δ: 8.34 (d, *J* = 7.2 Hz, 4H, naphthalene-H), 8.08 (d, *J* = 8.0 Hz, 4H, naphthalene-H), 7.62 (t, *J* = 7.8 Hz, 4H, naphthalene-H), 5.56 (q, *J* = 5.04 Hz, 2H, BocNHCH), 3.19 (s, 4H, NCH_2_), 2.93 (t, *J* = 7.8 Hz, 4H, CHCH_2_), 2.07–2.22 (m, 4H, NCH_2_CH_2_), 1.68–1.72 (m, 4H, CHCH_2_CH_2_), 1.53–1.55 (m, 4H, CONHCH_2_CH_2_), 1.21–1.54 (m, 4H, CH_2_CH_2_). ^13^C-NMR (100 MHz, D_2_O) δ: 172.39, 171.06, 165.59, 135.68, 131.94, 130.93, 127.24, 127.04, 120.61, 54.07, 42.79, 39.12, 38.91, 27.27, 26.39, 25.89, 25.68, 22.59. HR-MS *m*/*z* (ESI), Calcd for C_40_H_44_N_6_O_6_ [M + H]^+^ 705.3395, found found 705.3382.

*Bis-(N-epsilon-1,8-naphthalimido-lysyl)-1,6-hexylenediamine* (**4c**). Yield 83.6%. ^1^H-NMR (400 MHz, D_2_O) δ: 8.17 (d, *J* = 7.2 Hz, 4H, naphthalene-H), 8.05 (d, *J* = 8.0 Hz, 4H, naphthalene-H), 7.49 (t, *J* = 7.8 Hz, 4H, naphthalene-H), 5.20 (q, *J* = 5.6 Hz, 2H, BocNHCH), 2.78–2.92 (m, 4H, NCH_2_, CHCH_2_), 1.89–2.13 (m, 4H, NCH_2_CH_2_), 1.61–1.70 (m, 4H, CHCH_2_CH_2_), 1.23–1.35 (m, 4H, NHCH_2_CH_2_), 1.05 (d, *J* = 5.2 Hz, 4H, CONHCH_2_CH_2_), 0.80 (t, *J* = 6.2 Hz, 4H, CONHCH_2_CH_2_CH_2_). ^13^C-NMR (100 MHz, D_2_O) δ: 170.96, 165.32, 135.37, 131.41, 130.97, 127.26, 126.94, 120.86, 54.16, 39.17, 38.41, 28.09, 27.52, 26.46, 24.89, 22.85. HR-MS *m*/*z* (ESI), Calcd for C_42_H_48_N_6_O_6_ [M + H]^+^ 733.3708, found 733.3665.

*Bis-(N-epsilon-1,8-naphthalimido-lysyl)-1,4-phenylenedimethan-amine* (**4d**). Yield 78.9%. ^1^H-NMR (400 MHz, D_2_O) δ: 7.77–7.88 (m, 4H, naphthalene-H), 7.50–7.77 (m, 4H, naphthalene-H), 7.25–7.49 (m, 4H, naphthalene-H), 6.98–7.04 (m, 4H, benzene-H), 7.02 (m, 4H, benzene-H), 5.37 (t, *J* = 7.0 Hz, 2H, BocNHCH), 3.85–4.45 (m, 4H, xylyl-H), 2.92–2.98 (m, 4H, NCH_2_), 1.92–2.24 (m, 4H, CHCH_2_), 1.67–1.77 (m, 4H, NCH_2_CH_2_), 1.26–1.39 (m, 4H, CHCH_2_CH_2_). ^13^C-NMR (100 MHz, CDCl_3_) δ: 171.33, 165.03, 137.90, 135.17, 131.10, 130.50, 128.43, 126.73, 126.65, 120.23, 57.41, 54.21, 43.02, 39.22, 27.63, 26.54, 25.89, 23.03. HR-MS *m*/*z* (ESI), Calcd for C_44_H_44_N_6_O_6_ [M + H]^+^ 753.3395, found 753.3312.

*Bis-(N-epsilon-1,8-naphthalimido-lysyl)-1,3-phenylenedimethan-amine* (**4e**). Yield 88.9. ^1^H-NMR (400 MHz, D_2_O) δ: 7.88 (d, *J* = 7.2 Hz, 4H, naphthalene-H), 7.77 (d, *J* = 8.0 Hz, 4H, naphthalene-H), 7.24 (t, *J* = 7.8 Hz, 4H, naphthalene-H), 6.98–7.04 (m, 4H, benzene-H), 5.35 (q, *J* = 6.04 Hz, 2H, BocNHCH), 4.05–4.23 (q, *J* = 15.04 Hz, 4H, xylyl-H), 2.97 (t, *J* = 7.6 Hz, 4H, NCH_2_), 1.94–2.22 (m, 4H, CHCH_2_), 1.69–1.76 (m, 4H, NCH_2_CH_2_), 1.32–1.39 (m, 4H, CHCH_2_CH_2_). ^13^C-NMR (100 MHz, CDCl_3_) δ: 171.33, 165.03, 137.90, 135.17, 131.10, 130.50, 128.43, 126.73, 126.65, 120.23, 57.41, 54.21, 43.02, 39.22, 27.63, 26.54, 25.89, 23.03. HR-MS *m*/*z* (ESI), Calcd for C_44_H_44_N_6_O_6_ [M + H]^+^ 753.3395, found 753.3358.

### 3.2. UV–Vis Titration

A quantity of 20 μL stock solution of each bis-naphthalimide compound **4a**–**e** (5 mM) was diluted with 3 mL phosphate buffer (100 mM, pH = 7.4). An increasing volume of CT-DNA solution was added into the solution. Then, the solution was stirred and incubated at 25 °C for 10 min. The UV–Vis spectra in the absence and presence of DNA were recorded using a Shimadzu UV-2501 spectrophotometer. The binding constant *K_b_* was calculated from a *D*/*Δε_app_* vs. *D* plot according to the following equation [31]:

*D*/*Δε_app_* = *D*/*Δε* + 1/[(*Δε*)*K_b_*]

where *D* is the concentration of DNA, *Δε_app_* = [*ε_A_ − ε_F_*], ε_A_ = *A_obs_*/[compound], *Δε* = [*ε_B_ − ε_F_*], and *ε_B_* and *ε_F_* correspond to the extinction coefficients of the DNA–compound adduct and unbound compound, respectively.

### 3.3. Viscosity Study

Viscometric titration was performed at 25 °C using an Ubblehode viscometer. A quantity of 5 mL CT DNA (2.8 mM) was diluted with 20 mL phosphate buffer (50.0 mM, pH 7.4). The different flow time was measured using a stopwatch while varying the concentrations of compounds. The plot of (*η*/*η*_0_)^1/3^ vs. *r* was obtained according to the flow time, where *η* and *η*_0_ are the flow time of the presence and absence of compounds, respectively, and *r* is equal to [compound]/[DNA].

### 3.4. Cytotoxicity Assay

Human gastric carcinoma cell line (BGC823) and human esophageal carcinoma cell line (EC109) were cultured in RPMI 1640 medium supplemented with 10% fetal bovine serum, 100 units/mL penicillin, and 100 units/mL penicillin streptomycin. The cell culture was kept in 5% CO_2_ under humidified conditions at 37 °C. The culture solution was changed every other day, and the subcultures were performed with 0.25% trypsin. The compounds were solubilized in RPMI 1640 medium and diluted to different concentrations immediately prior to use.

The cytotoxicity of bis-naphthalimide compounds was assessed using the thiazolyl blue tetrazolium bromide (MTT) assay in vitro. Tumor cells were planted into 96-well microtiter plates at a density of 5.0 × 10^4^ cells/well. After being cultured in 5% CO_2_ under humidified conditions at 37 °C for 24 h, various compound medium solutions were added to obtain the final drug concentrations of 0, 12.5, 25, 50, 100, and 200 μmol/L, respectively. After incubation for 24 h, 10 μL MTT (5 mg/L) was added to each cell and the mixture was incubated for 4 h. Then, the medium was removed and replaced by 150 μL DMSO to solubilize the converted purple dye in the culture plates. The absorbance of each cell was measured using a Bio-rad 680 microplate reader (Hercules, CA, USA) at 490 nm. The IC_50_, which inhibits the growth of 50% of cells relative to nontreated control cells, was calculated as the concentration of the tested compound by linear fitting.

### 3.5. Morphology Observation

Tumor cells were planted into 24-well microtiter plates at a density of 5.0 × 10^6^ cells/well. After incubation for 24 h, compound solution was added and incubated for 48 h. Morphological changes of the cells were observed using light microscopy.

## 4. Conclusions

In this paper, a series of bis-naphthalimide derivatives with different diamine linkers were designed and synthesized. The DNA binding constants and binding modes of the compounds were measured by UV–Vis titration and viscosity experiments. The results showed that the length of diamine linkers significantly influenced the binding ability of the bis-naphthalimide derivatives. The compounds with shorter linkers showed larger binding constants and the classical binding mode. However, in cytotoxicity assay experiments, the compound **4d** with the rigid *p*-xylylenediamine linker showed better cytotoxicity than did the other bis-naphthalimide derivatives. The morphology observation of BGC823 cell line incubated with **4d** also exhibited the inhibition of cancer cell growth.

## Figures and Tables

**Table 1:** The *K_b_* values of compounds **4a**–**e** with CT DNA.

Compound	*K_b_* (M^−1^)
**4a**	3.40 × 10^4^
**4b**	2.78 × 10^4^
**4c**	1.54 × 10^4^
**4d**	7.29 × 10^3^
**4e**	1.00 × 10^4^

**Table 2:** *K_b_* values for compound **4a** with various DNA sequences.

DNA Duplex	*K_b_* (M^−1^)
[Poly(dA)-Poly(dT)]	9.08 × 10^3^
[Poly(dG)-Poly(dC)]	1.15 × 10^3^
AT Box	9.71 × 10^3^
GC Box	1.91 × 10^3^

**Table 3:** The cytotoxicity of compounds **4a**–**e**.

Cell Lines	IC_50_ Value (μmol∙L^−1^)					
	Fluorouracil	4a	4b	4c	4d	4e
EC109	45.82	694.59	456.35	330.38	142.45	354.48
BGC823	49.88	N.T. ^1^	N.T.	193.65	77.99	N.T.

^1^ N.T.: not tested.
